# mRNA vaccination drives differential mucosal neutralizing antibody profiles in naïve and SARS-CoV-2 previously-infected individuals

**DOI:** 10.3389/fimmu.2022.953949

**Published:** 2022-09-08

**Authors:** Stephanie Longet, Alexander Hargreaves, Saoirse Healy, Rebecca Brown, Hailey R. Hornsby, Naomi Meardon, Tom Tipton, Eleanor Barnes, Susanna Dunachie, Christopher J. A. Duncan, Paul Klenerman, Alex Richter, Lance Turtle, Thushan I. de Silva, Miles W. Carroll

**Affiliations:** ^1^ Wellcome Centre for Human Genetics, University of Oxford, Oxford, United Kingdom; ^2^ Pandemic Sciences Institute, University of Oxford, Oxford, United Kingdom; ^3^ Department of Infection, Immunity and Cardiovascular Disease, University of Sheffield, Sheffield, United Kingdom; ^4^ Sheffield Teaching Hospitals NHS Foundation Trust, Sheffield, United Kingdom; ^5^ Nuffield Department of Clinical Medicine, Peter Medawar Building for Pathogen Research, University of Oxford, Oxford, United Kingdom; ^6^ Oxford University Hospitals NHS Foundation Trust, Oxford, United Kingdom; ^7^ Nuffield Department of Clinical Medicine, Oxford Centre For Global Health Research, University of Oxford, Oxford, United Kingdom; ^8^ Mahidol-Oxford Tropical Medicine Research Unit, Mahidol University, Bangkok, Thailand; ^9^ Translational and Clinical Research Institute Immunity and Inflammation Theme, Newcastle University, Newcastle, United Kingdom; ^10^ Department of Infection and Tropical Medicine, Newcastle upon Tyne Hospitals NHS Foundation Trust, Newcastle, United Kingdom; ^11^ Translational Gastroenterology Unit, University of Oxford, Oxford, United Kingdom; ^12^ NIHR Oxford Biomedical Research Centre, University of Oxford, Oxford, United Kingdom; ^13^ Institute of Cancer and Genomic Science, College of Medical and Dental Science, University of Birmingham, Birmingham, United Kingdom; ^14^ University Hospitals Birmingham NHS Foundation Trust, Birmingham, United Kingdom; ^15^ NIHR Health Protection Research Unit in Emerging and Zoonotic Infections, Institute of Infection, Veterinary and Ecological Sciences, University of Liverpool, Liverpool, United Kingdom; ^16^ Liverpool University Hospitals NHS Foundation Trust, Liverpool, United Kingdom

**Keywords:** antibody responses, mucosal immunity, SARS-CoV-2, mRNA vaccination, neutralizing responses, ACE2 inhibition

## Abstract

Two doses of BNT162b2 mRNA vaccine induces a strong systemic SARS-CoV-2 specific humoral response. However, SARS-CoV-2 airborne transmission makes mucosal immune response a crucial first line of defense. Therefore, we characterized SARS-CoV-2-specific IgG responses induced by BNT162b2 vaccine, as well as IgG responses to other pathogenic and seasonal human coronaviruses in oral fluid and plasma from 200 UK healthcare workers who were naïve (N=62) or previously infected with SARS-CoV-2 (N=138) using a pan-coronavirus multiplex binding immunoassay (Meso Scale Discovery^®^). Additionally, we investigated the impact of historical SARS-CoV-2 infection on vaccine-induced IgG, IgA and neutralizing responses in selected oral fluid samples before vaccination, after a first and second dose of BNT162b2, as well as following a third dose of mRNA vaccine or breakthrough infections using the same immunoassay and an ACE2 inhibition assay. Prior to vaccination, we found that spike-specific IgG levels in oral fluid positively correlated with IgG levels in plasma from previously-infected individuals (Spearman r=0.6858, p<0.0001) demonstrating that oral fluid could be used as a proxy for the presence of plasma SARS-CoV-2 IgG. However, the sensitivity was lower in oral fluid (0.85, 95% CI 0.77-0.91) than in plasma (0.94, 95% CI 0.88-0.97). Similar kinetics of mucosal and systemic spike-specific IgG levels were observed following vaccination in naïve and previously-infected individuals, respectively. In addition, a significant enhancement of OC43 and HKU1 spike-specific IgG levels was observed in previously-infected individuals following one vaccine dose in oral fluid (OC43 S: p<0.0001; HKU1 S: p=0.0423) suggesting cross-reactive IgG responses to seasonal beta coronaviruses. Mucosal spike-specific IgA responses were induced by mRNA vaccination particularly in previously-infected individuals (71%) but less frequently in naïve participants (23%). Neutralizing responses to SARS-CoV-2 ancestral and variants of concerns were detected following vaccination in naïve and previously-infected participants, with likely contribution from both IgG and IgA in previously-infected individuals (correlations between neutralizing responses and IgG: Spearman r=0.5642, p<0.0001; IgA: Spearman r=0.4545, p=0.0001). We also observed that breakthrough infections or a third vaccine dose enhanced mucosal antibody levels and neutralizing responses. These data contribute to show that a previous SARS-CoV-2 infection tailors the mucosal antibody profile induced by vaccination.

## Introduction

Severe acute respiratory syndrome coronavirus 2 (SARS-CoV-2), the causative agent of coronavirus disease 2019 (COVID-19) (1), has led to the death of more than 6.4 million individuals worldwide (2). The rapid development and use of various COVID-19 vaccines provide an efficient tool to prevent severe disease (3). Licensed COVID-19 vaccines were shown to induce strong systemic humoral and cellular immune responses (4, 5). However, SARS-CoV-2 primarily uses the respiratory epithelium as its route of entry (6), in addition to the oral mucosa (7) and conjunctival surfaces (8). Its respiratory transmission makes mucosal immunity a crucial first line of defense. The study of oral fluid samples is an easy and non-invasive way to analyze mucosal antibody responses following infection and vaccination. The analysis of this biological fluid can provide precious information on antibody quantity, kinetics, isotype and neutralizing responses at mucosal surfaces. An essential component of mucosal humoral immunity is IgA which was shown to play an important role in host defense against respiratory viruses (9). Secretory IgA (dimer/polymer) (SIgA) can be induced at mucosal sites but monomeric IgA can also leak from the circulatory compartment to mucosal surfaces (10). In addition, IgG can reach mucosal surfaces from the blood (10). Thus, the level of IgG in oral fluid can mirror the level of systemic IgG, which highlights the potential application of oral fluid sampling as an alternative to serum samples for large scale serological studies to determine immune status of populations to a specific pathogen.

Oral fluid-based assays have been shown to be an effective method to measure SARS-CoV-2 antibody response after infection (11–16) and administration of SARS-CoV-2 mRNA-based vaccines (16–18). These studies showed that SARS-CoV-2 spike-specific IgG was able to persist in saliva (16), while spike-specific IgA and IgM rapidly declined over time following infection (12, 13). However, Sterlin et al. reported that SARS-CoV-2-specific IgA responses dominated neutralizing responses early post-infection (15). Following mRNA vaccination, Ketas et al. detected anti-spike IgG in all saliva samples from naïve individuals after two doses, while anti-spike IgA was only detected in 59% of BNT162b2 vaccine recipients (17). This study suggests that both vaccine-induced IgG and IgA had likely transuded from the blood into mucosal sites (17).

In our study, we analyzed antibody responses in oral fluid samples from a cohort of UK healthcare workers (HCWs) who were naïve (N=62) or previously-infected with SARS-CoV-2 (N=138). The first objective was to evaluate the sensitivity and specificity of our multiplex MSD^®^ ELISA to detect SARS-CoV-2-specific IgG in oral fluid versus in plasma. The second objective was to compare the kinetics of SARS-CoV-2-specific IgG responses induced by BNT162b2 vaccine in oral fluid and plasma side-by-side, as well as mucosal and systemic IgG responses specific to other pathogenic and seasonal human coronaviruses. The third objective was to evaluate the impact of a previous SARS-CoV-2 infection on vaccine-induced mucosal IgG and IgA profiles and neutralizing responses following one, two and three doses of mRNA vaccine, or after a breakthrough infection.

## Materials and methods

### Study and participants

This study is part of the Protective Immunity from T-cells in HCWs (PITCH) consortium, which is a prospective, observational, cohort study, with HCWs recruited from various sites. HCWs from Sheffield Teaching Hospitals NHS Foundation Trust were recruited by personal communication, hospital e-mail communications and from hospital-based staff SARS-CoV-2 screening programs. Eligible participants were adults aged ≥18 currently working as HCWs, including allied support and laboratory staff. Participants received SARS-CoV-2 vaccinations *via* the national immunization program and not as part of a study protocol. At Sheffield Teaching Hospitals NHS Foundation Trust, participants were recruited under the Sheffield Teaching Hospitals Observational Study of Patients with Pulmonary Hypertension, Cardiovascular and other Respiratory Diseases (STH-Obs; 18/YH/0441), which was amended for this study on September 10 2020. PITCH was recognized as a sub-study of SARS-CoV-2 Immunity and Reinfection Evaluation (SIREN) Study on the 2^nd^ of December 2020, which is a large multi-center prospective cohort study of HCWs in NHS hospitals in the UK and which was approved by the Berkshire Research Ethics Committee, Health Research 250 Authority (IRAS ID 284460, REC reference 20/SC/0230). The study was conducted in compliance with the principles of the Declaration of Helsinki (2008) and the International Conference on Harmonization (ICH) Good Clinical Practice (GCP) guidelines.

Two hundred participants who provided oral fluid and plasma on at least one timepoint (baseline, post-1^st^ BNT162b2 dose, post-2^nd^ BNT162b2 dose or post-3^rd^ BNT162b2 or mRNA-1273 dose) were included in this study between December 2020 and February 2022. Individuals were defined as SARS-CoV-2 naïve or previously-infected based on documented PCR and/or serology results from NHS serology testing. Previously-infected individuals experienced mild or asymptomatic SARS-CoV-2 infection. Baseline sampling was performed at a median of 216 days (IQR 111.0-245.0) days following a SARS-CoV-2 positive PCR result. Post-vaccine immune responses were assessed at a median of 28 days after first dose (IQR 26-32) and second dose (IQR 26-32). Participants received the 1^st^ and the 2^nd^ dose with a dosing interval defined as a long interval (> 5 weeks) except 1 naïve and 3 previously-infected individuals. The median dosing interval between the 1^st^ and 2^nd^ dose was 63 days (IQR 63-69). Immune responses were also measured in a subset of participants at a median of 28 days (IQR 27-43) after the 3^rd^ dose, with a median dosing interval between the 2^nd^ and 3^rd^ dose of 214 days (IQR 200-235) (**Table 1**). Immune responses were analyzed between 16 to 28 days following a breakthrough infection post-2^nd^ dose in seven participants including two participants who were vaccinated twice with AstraZeneca vaccine (AZ). Individuals vaccinated with AZ were only included in antibody analysis following a breakthrough infection and not in the other analyses.

**Table 1:** Characteristics of healthcare workers included in the study. Values are shown in median + interquartile range.

	Naïve	Previously-infected
**N**	62	138
**Age in years (IQR)**	43.5 (34.0-51.0)	49.0 (38.0-55.0)
**Female (%)** **Male (%)**	52 (83.9%) 10 (16.1%)	120 (87.0%) 18 (13.0%)
**Days from infection to baseline sampling (IQR)**	–	216.0 (111.0-245.0)
**Days from first dose to second sampling (IQR)**	28.0 (26.0-32.0)	28.5 (26.0-32.0)
**Days from second dose to third sampling (IQR)**	28.0 (25.0-32.0)	28.5 (26.0-31.5)
**Days from third dose to fourth sampling (IQR)**	31.5 (26.0-36.5)	32.0 (27.0-53.0)
**Vaccine interval days first to second dose (IQR)**	63.0 (63.0-67.0)	63.0 (62.0-69.0)
**Vaccine interval days second to third dose (IQR)**	208.0 (198.0-235.0)	216.0 (200.0-235.0)

### Sampling and processing of oral fluid samples

Oral fluid was collected from participants prior to vaccination where possible (“unvaccinated”), after the first dose (“post-1^st^”), after the second dose (“post-2^nd^”) and after the third dose (“post-3^rd^”). Oral fluid was also collected from seven participants who experienced a SARS-CoV-2 breakthrough infection between the 2^nd^ and the 3^rd^ dose.

Oral fluid samples were collected in an Oracol+ swab (Malvern Medical Developments). Saliva and gingival crevicular fluid were collected with these swabs. Samples were processed within 6 hours of collection. The samples were centrifuged at 1,300 x g for 10 minutes at 4°C. A storage buffer composed of 10% v/v Fetal Calf Serum (FCS) in Dulbecco’s Phosphate buffered saline supplemented with 0.5% v/v Gentamycin (50 mg/ml stock), 0.2% v/v Fungizone (250 ug/ml stock), 11 mg/l sterile Red dye Phenol red solution (0.5% w/v stock) and 1 x protease inhibitor cocktail (Calbiochem, Cat No 539131) was added at a 1:1 ratio to oral fluid prior to storage in aliquots.

### Sampling and processing of plasma samples

Blood was collected from HCWs prior to vaccination where possible (“unvaccinated”), after the first dose (“post-1^st^”), after the second dose (“post-2^nd^”) and after the third dose (“post-3^rd^”). Blood was also collected from seven participants who experienced a SARS-CoV-2 breakthrough infection between the 2^nd^ and the 3^rd^ dose.

Up to 70ml of blood were collected at each timepoint over a period of 3-14 months. Peripheral blood mononuclear cells (PBMCs) were separated from heparinized or Ethylenediaminetetraacetic acid (EDTA) whole blood using density gradient centrifugation. Freshly isolated PBMCs were used in other studies. Extracted plasma was stored at -80°C until antibody analysis.

### Meso scale discovery IgG and IgA binding assays

IgG and IgA responses to SARS-CoV-2, SARS-CoV-1, MERS and seasonal coronaviruses were measured using a multiplexed MSD^®^ immunoassay: The V-PLEX COVID-19 Coronavirus Panel 3 (IgG) Kit (cat. no. K15399U and K15401U) from Meso Scale Diagnostics, Rockville, MD USA. A MULTI-SPOT^®^ 96-well, 10 spot plate was coated with three Wuhan SARS-CoV-2 antigens (Spike (S), Receptor-Binding Domain (RBD), Nucleoprotein (N), SARS-CoV-1 and MERS spike trimers, as well as spike proteins from seasonal human coronaviruses, OC43, HKU1, 229E and NL63, and bovine serum albumin. Antigens were spotted at 200−400 μg/mL (MSD^®^ Coronavirus Plate 3). Multiplex MSD^®^ assays were performed as per the instructions of the manufacturer. To measure IgG or IgA binding antibodies, 96-well plates were blocked with MSD^®^ Blocker A for 30 minutes. Following washing with washing buffer, oral samples diluted 1:10-1:100 and plasma samples diluted 1:1,000-10,000 in diluent buffer and added to wells, along with MSD^®^ standard or undiluted MSD^®^ internal controls. After a 2-hour incubation and a washing step, detection antibody (MSD SULFO-TAG™ Anti-Human IgG or IgA Antibody, 1/200) was added. Following washing, MSD GOLD™ Read Buffer B was added and plates were read using a MESO^®^ SECTOR S 600 Reader. The standard curve was established by fitting the signals from the standard using a 4-parameter logistic model. Concentrations of samples were determined from the electrochemiluminescence signals by back-fitting to the standard curve and multiplied by the dilution factor. Concentrations are expressed in Arbitrary Units/ml (AU/ml). Cut-offs for plasma and oral fluids were determined for each SARS-CoV-2 antigen (S, RBD and N) based on SARS-CoV-2 naïve individual samples shown to be SARS-CoV-2 seronegative using NHS diagnostic laboratory serological tests (“unvaccinated naïve”) (average concentration + 3xStandard Deviation for IgG binding and average concentration + 1xStandard Deviation for IgA binding). As samples were from UK individuals with low probability to have been exposed to SARS-CoV-1 and MERS, cut-offs for SARS-CoV-1 S and MERS S were similarly determined (**Table 2**).

**Table 2:** Cut-offs for plasma and oral fluid samples determined for the MSD^®^ IgG and IgA immunoassays.

Antigens	Cut-offs for plasma		Cut-offs for oral fluid	
	IgG	IgA	IgG	IgA
SARS-CoV-2 S	542 AU/ml	288 AU/ml	1.88 AU/ml	28.35 AU/ml
SARS-CoV-2 RBD	2348 AU/ml	—	5.39 AU/ml	31.82 AU/ml
SARS-CoV-2 N	9948 AU/ml	—	16.56 AU/ml	50.75 AU/ml
SARS-CoV-1 S	4542 AU/ml	—	1.80 AU/ml	14.41 AU/ml
MERS S	910 AU/ml	—	2.54 AU/ml	253.82 AU/ml

The dilutions of plasma and oral fluid samples were determined by preliminary experiments in order to be in the range of the MSD^®^ standard concentrations.

### Meso scale discovery ACE2 inhibition surrogate neutralization assay

An alternative MSD^®^ immunoassay (V-PLEX SARS-CoV-2 Panel 13 (ACE-2) Kit cat numbers** **K15466U or Panel 23 Kit cat no. K15570U) was used to measure the ability of oral fluid samples to inhibit angiotensin-converting enzyme 2 (ACE2) binding to different variants of SARS-CoV-2 spike including B lineage Wuhan-Hu-1 spike (WT), B.1.1.7/Alpha, B.1.617.2/Delta, B.1.351/Beta, P.1/Gamma and Omicron BA.1. Assays were performed as per manufacturer’s instructions with neat oral fluid samples. To measure ACE2 inhibition, 96-well MSD^®^ plates were blocked with MSD^®^ Blocker for 30 minutes. Plates were then washed in MSD^®^ washing buffer, and 25 μl of undiluted oral fluid samples were added to the plate (original sample diluted 1:2 in storage buffer as detailed above). After 1-hour incubation, recombinant human ACE2-SULFO-TAG™ was added to all wells. After a further 1-hour, plates were washed and MSD GOLD™ Read Buffer B was added, plates were then immediately read using a MESO^®^ SECTOR S 600 Reader. Neutralizing activity was determined by measuring the presence of antibodies able to block the binding of ACE2 to SARS-CoV-2 spike proteins from Wuhan-Hu-1 spike, B.1.1.7/Alpha, B.1.617.2/Delta, B.1.351/Beta, P.1/Gamma and Omicron BA.1 was expressed as percentage of ACE2 inhibition in comparison to the blanks on the same plate.

### Statistical analysis

The cohort size was determined by the total number of HCWs recruited to the study rather than a pre-determined sample size calculation. Paired comparisons before and after vaccination were performed using the Wilcoxon matched pairs signed-rank test. Unpaired comparisons across two groups were performed using the Mann-Whitney test. Pairwise correlations were assessed using Spearman’s rank-order correlation (r). Correlation coefficients were interpreted as low (r=0·20–0·49), moderate (r=0·50–0·69), high (r=0·70–0·89), or very high (r=0·90–1·00) with a p<0.05. The median, mean and 95% interquartile range (IQR) were estimated for binding antibody levels. A threshold of p<0.05 was used to define a statistically significant result. Statistical analyses were done using GraphPad Prism 9.2.0. The sensitivity and specificity of the assays were determined using the Wilson-Brown method of Graph Pad 9.2.0. STATA 17.0 was used for the statistics linked to the **Table 1** showing the characteristics of the cohort.

## Results

### Vaccine-induced SARS-CoV-2-specific IgG response strongly correlates in oral fluid and plasma

Plasma and oral fluid were collected from 200 SARS-CoV-2 naïve or previously-infected HCWs between December 2020 and February 2022 on at least one following timepoint (baseline, post-1^st^ dose, post-2^nd^ dose) and also post-3^rd^ dose in a subset of participants (**Table 1**). The median age of the cohort was 47 years (IQR 37-54) and 86% were female. Post-vaccine immune responses were assessed at a median of 28 days after each dose (**Table 1**).

**Figure 1:**
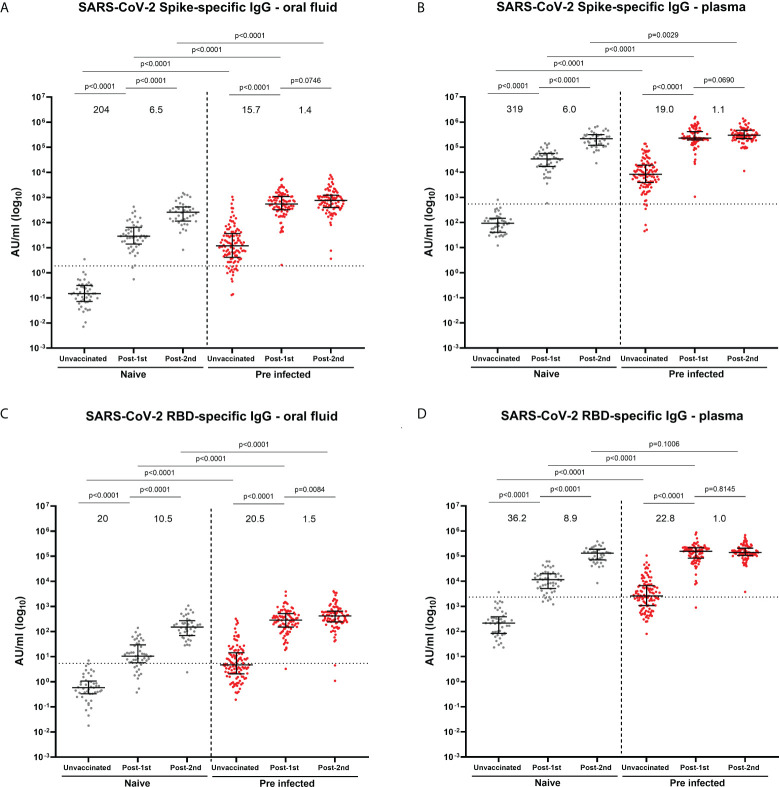
SARS-CoV-2 S- and RBD-specific IgG concentrations in oral fluid and plasma from naïve and previously-infected individuals analyzed by multiplex MSD^®^ assay. **(A)** S-specific IgG in oral fluid and **(B)** in plasma; **(C)** RBD-specific IgG in oral fluid and **(D)** in plasma. Data are shown in concentrations expressed in Arbitrary Units/ml (AU/ml). Mann-Whitney tests were used to determine the statistical significance between the groups of samples. Values above the columns show the fold increases in S- and RBD-specific IgG levels. Dashed lines show the cut-offs based on IgG responses in 45 unvaccinated naïve samples (average concentration + 3SD).

Concentrations of SARS-CoV-2 spike-specific IgG were measured in oral fluid and plasma before vaccination and after the 1^st^ and 2^nd^ vaccine dose (**Figure 1**; **Supplementary Figures 1, 2**). Prior to vaccination, 85.2% and 94.4% of SARS-CoV-2 previously-infected HCWs were positive for S-specific IgG in oral fluid and plasma, respectively (**Figures 1A, B**). Only 47.2% and 53.7% of previously-infected individuals were positive for RBD-specific IgG in oral fluid and plasma, respectively (**Figure 1C, D**). The majority of previously-infected individuals were negative for N-specific IgG in both oral fluid and plasma (**Supplementary Figures 1A, B**). Significant positive correlations were seen between S-, RBD- and N-specific IgG levels measured in oral fluid and plasma from previously-infected individuals before vaccination (S: Spearman r=0.6858, p<0.0001; RBD: Spearman r=0.6543, p<0.0001; N: Spearman r=0.7117, p<0.0001) (**Supplementary Figures 3A–C**).

To analyze the sensitivity and specificity of the MSD^®^ immunoassay, we compared S-specific IgG responses between oral fluid and plasma from previously-infected and naïve HCWs before vaccination (**Supplementary Figure 3D**). The specificity of the MSD^®^ multiplex immunoassay was the same in oral fluid and plasma (0.98, 95% CI 0.88-1.00 in both compartments). However the sensitivity was lower in oral fluid than in plasma (0.85, 95% CI 0.77-0.91 vs 0.94, 95% CI 0.88-0.97) (**Table 3**). Similar sensitivity estimates were observed using only the previously-infected individuals confirmed by PCR test (oral fluid: 0.89, 95% CI 0.81-0.95; plasma: 0.97, 95% CI 0.91-1.00) (**Supplementary Table 1**).

**Table 3:** Sensitivity and specificity of MSD^®^ immunoassay with 95% confidence intervals.

Oral fluid	S-specific IgG	No S-specific IgG	Sensitivity (95% CI)	Specificity (95% CI)
Previously-infected	92	16	0.85 (0.77-0.91)	0.98 (0.88-1.00)
Naive	1	44		
Plasma	S-specific IgG	No S-specific IgG	Sensitivity (95% CI)	Specificity (95% CI)
Previously-infected	102	6	0.94 (0.88-0.97)	0.98 (0.88-1.00)
Naive	1	44		

Analysis performed using S-specific IgG responses measured in oral fluid and plasma samples from previously-infected HCWs defined by PCR and/or NHS serology and Naïve individuals at pre-vaccination stage. Previously-infected individuals N=108, Naïve individuals N=45.

An increase in S- and RBD-specific IgG was observed following BNT162b2 mRNA vaccination in both oral fluid and plasma in naïve and previously-infected participants (**Figures 1A–D**; **Supplementary Figure 2A–D**). In naïve individuals, the first dose elicited 204-fold and 319-fold increases in S-specific IgG levels in oral fluid and plasma respectively, compared to 15.7-fold and 19.0-fold increases in previously-infected individuals (**Figures 1A, B**). Following the second dose, further increases in S-specific IgG levels were seen in oral fluid (naïve: 6.5-fold; previously-infected 1.4-fold) and plasma (infection-naïve: 6.0-fold; previously-infected 1.1-fold) (**Figures 1A, B**). The dynamics of RBD-specific IgG responses were similar to S-specific IgG responses following each vaccine dose (**Figures 1C, D**). Overall, vaccine-induced S- and RBD-specific IgG levels were significantly higher in both oral fluid and plasma from SARS-CoV-2 previously-infected compared to naïve HCWs. As expected, vaccination did not affect the systemic and mucosal N-specific response (**Supplementary Figures 1A, B; Supplementary Figures 2E, F**).

**Figure 2:**
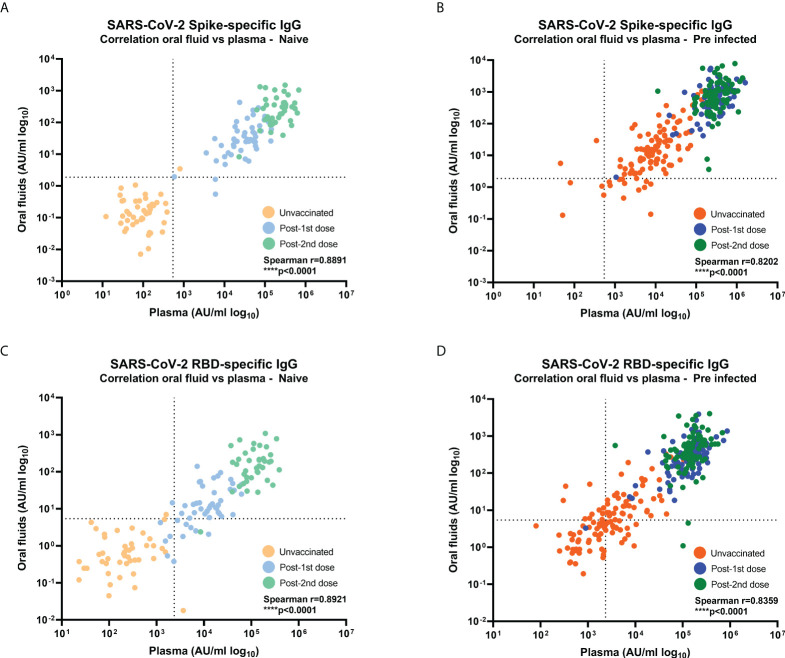
Correlations between SARS-CoV-2-specific IgG levels in oral fluid versus plasma from naïve and previously-infected individuals. Concentrations (AU/ml) of **(A, B)** S-specific IgG and **(C, D)** RBD-specific IgG were determined by MSD^®^ multiplex immunoassay in naïve **(A, C)** and previously-infected **(B, D)** HCWs. Pairwise correlations were assessed using Spearman’s rank-order correlation. Dashed lines show the cut-offs based on IgG responses in 45 unvaccinated naïve samples (average concentration + 3SD).

Combining data from all timepoints, S- and RBD-specific IgG responses measured by MSD^®^ immunoassay in oral fluids highly correlated with those in plasma samples from naïve and previously-infected individuals (Naïve: S-specific IgG r=0.8891, p<0.0001 and RBD-specific IgG r=0.8921, p<0.0001; Previously-infected: S-specific IgG: r=0.8202, p<0.0001 and RBD-specific IgG r=0.8359, p<0.0001) (**Figures 2A–D**).

### Cross-reactive IgG responses to beta and pathogenic coronaviruses in oral fluid

**Figure 3:**
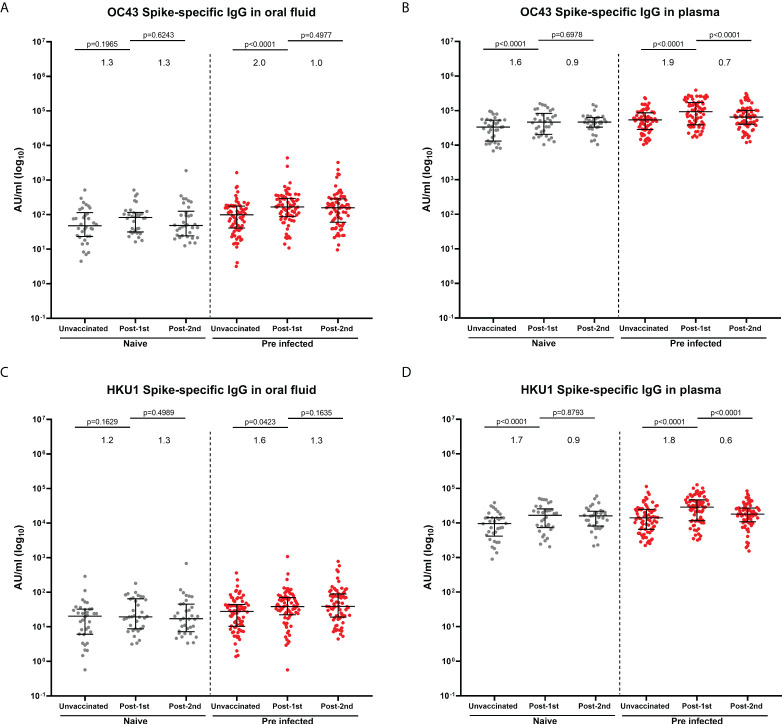
IgG responses to human seasonal beta coronaviruses in matched oral fluid and plasma. Concentrations (AU/ml) of OC43 **(A, B)** and HKU1 **(C, D)** S-specific IgG measured in oral fluid **(A, C)** and plasma **(B, D)** from naïve and previously-infected individuals using MSD^®^ multiplex immunoassay. Wilcoxon rank tests were used to determine the statistical differences between paired samples. Values above the columns show the fold increases in S-specific IgG levels. Matched naïve samples: N=34; Matched previously-infected samples: N=74.

In previous studies, we and others have reported that SARS-CoV-2 infection as well as a BNT162b2 dose boosted the pre-existing systemic IgG against human seasonal beta coronavirus spike proteins (OC43, HKU1), but not alpha coronavirus spike proteins (229E and NL63) in naïve and previously-infected individuals (4, 19). Therefore, we measured IgG responses to the four seasonal coronavirus spike proteins (OC43, HKU1, 229E and NL63) in oral fluid and plasma samples from the same individuals using the MSD^®^ multiplex immunoassay. IgG responses to four seasonal coronavirus spike proteins were detectable in oral fluid from naïve and previously-infected individuals before and after vaccination (**Figure 3**; **Supplementary Figure 4**). In samples from naïve individuals before vaccination, moderate correlations were observed between S-specific IgG levels measured in oral fluids and plasma (OC43 S: r=0.5581, p=0.0006; HKU1 S: r=0.6272, p<0.0001; 229E S: r=0.5954, p=0.0002; NL63S: r=0.4848, p=0.0037) (**Supplementary Figures 5A–D**).

In naïve individuals, one dose of BNT162b2 vaccine did not significantly enhance OC43- and HKU1-specific IgG in oral fluid (**Figures 3A, C**), whereas it significantly enhanced IgG against OC43 (p<0.0001) and HKU1 (p<0.0001) spike proteins in plasma (**Figures 3B, D**). A significant enhancement of OC43 and HKU1 S-specific IgG levels was observed in previously-infected individuals following one vaccine dose in oral fluid (OC43 S: p<0.0001; HKU1 S: p=0.0423) (**Figures 3A, C**) and plasma (OC43 S: p<0.0001; HKU1 S: p<0.0001) (**Figures 3B, D**). No increase in IgG against human seasonal alpha coronaviruses 229E (**Supplementary Figures 4A, B**) and NL63 (**Supplementary Figures 4C, D**) was detected either in oral fluid or in plasma following vaccination.

**Figure 4:**
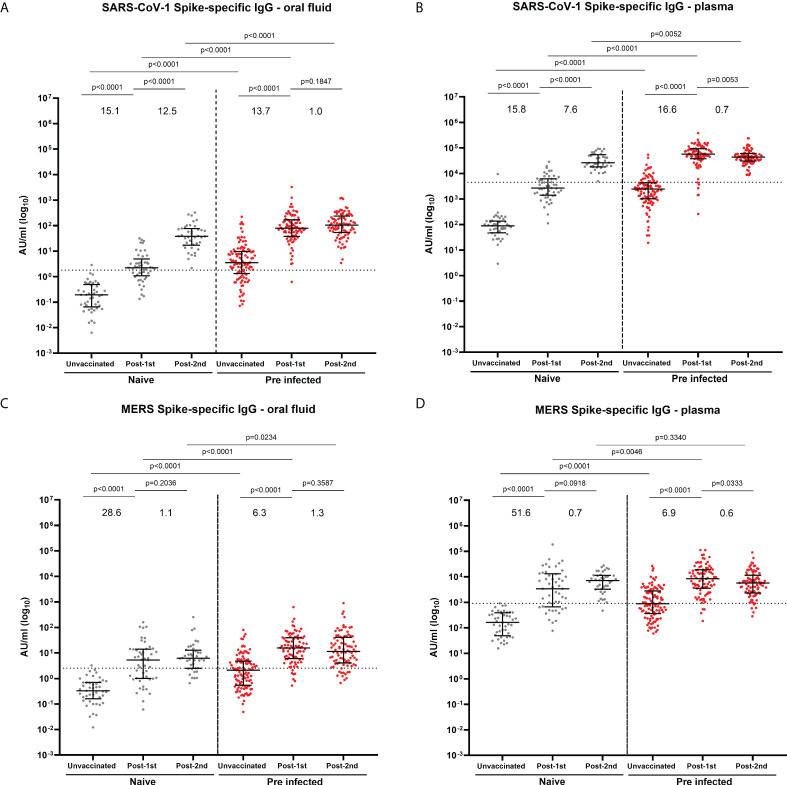
IgG responses to SARS-CoV-1 and MERS in oral fluid and plasma. Concentrations (AU/ml) of S-specific IgG to SARS-CoV-1 **(A, B)** and MERS S **(C, D)** in oral fluid **(A, C)** and plasma **(B, D)** from naïve and previously-infected individuals measured by multiplex MSD^®^ immunoassay. Values above the columns show the fold increases in S-specific IgG levels. Mann-Whitney tests were used to determine the statistical significance between the groups.

We also observed a significant induction of cross-reactive IgG to SARS-CoV-1 (**Figures 4A, B**) and MERS (**Figures 4C, D**) in oral fluid and plasma following infection and following one vaccine dose in both naïve and previously-infected individuals. A significant increase in mucosal and systemic IgG against SARS-CoV-1 was also detected following a second vaccine dose in naïve individuals (**Figures 4A, B**). Strong correlations were observed between IgG against SARS-CoV-1 (**Supplementary Figures 6A, B**) or MERS (**Supplementary Figures 6C, D**) in oral fluid versus plasma.

### SARS-CoV-2 previously-infected individuals generate SARS-CoV-2-specific IgA responses following mRNA vaccination

**Figure 5:**
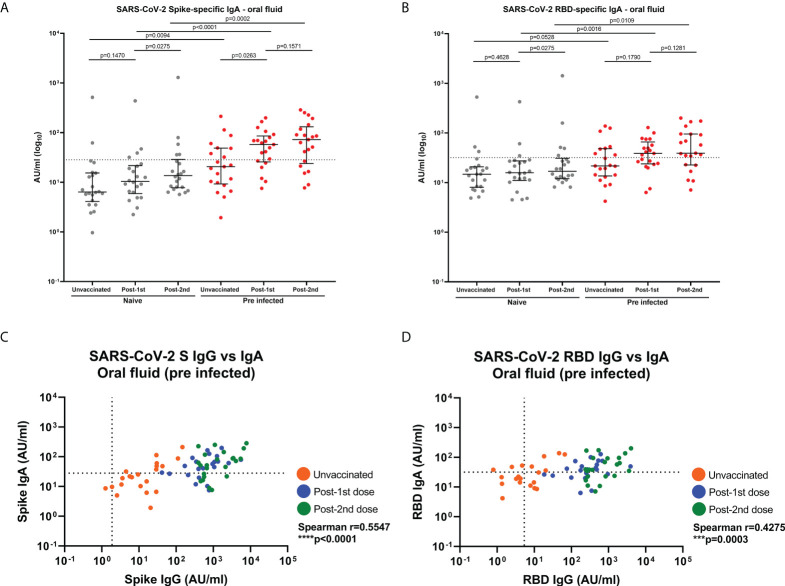
SARS-CoV-2 S- and RBD-specific IgA responses and correlations with IgG responses in oral fluid. SARS-CoV-2 specific IgA concentrations in matched oral fluid from naïve and previously-infected individuals determined by multiplex MSD^®^ immunoassay. **(A)** S-specific IgA and **(B)** RBD-specific IgA in oral fluid. Wilcoxon and Mann-Whitney tests were used to determine the statistical significance between the groups of paired and unpaired samples, respectively. Dashed lines show the cut-offs based on IgA responses in 22 unvaccinated naïve samples (average concentration +1SD). Correlations between SARS-CoV-2 S- **(C)** and RBD-specific **(D)** IgG versus IgA concentrations in oral fluid from previously-infected individuals. Pairwise correlations were assessed using Spearman’s rank-order correlation. ****=p-value <0.0001; ***=p-value <0.001.

As IgA is the most abundant antibody at mucosal surfaces, SARS-CoV-2-specific IgA responses were measured in oral fluid samples taken before and after vaccination in 22 naïve and 21 SARS-CoV-2 previously-infected individuals (**Figure 5**). Eight of the previously-infected (43%) and two naïve (9%) individuals showed S-specific IgA responses above the threshold before vaccination (**Figure 5A**). Following the first dose, a significant increase in S-specific IgA responses was seen in previously-infected individuals but not in naïve individuals (**Figure 5A**), with 15 (71%) of previously-infected showing detectable IgA above the threshold at this timepoint. The second vaccine dose did not increase S-specific IgA responses in previously-infected individuals further (**Figure 5A**). Only 5 (23%) naïve individuals showed S-specific IgA responses above our cut-off after the second dose (**Figure 5A**) compared to 15 (71%) of previously-infected individuals. A similar picture was seen for RBD-specific IgA responses (**Figure 5B**). Some individuals in naïve and previously-infected groups also showed N-specific IgA responses above our cut-off at each timepoint but as expected, S-based mRNA vaccination did not impact on N-specific IgA responses (**Supplementary Figure 7**). Significant low to moderate correlations between S- and RBD-specific IgG and IgA in oral fluid were seen in SARS-CoV-2 previously-infected individuals (S: Spearman r=0.5547, p<0.0001; RBD: Spearman r=0.4275, p=0.0003) (**Figures 5C, D**).

Interestingly, two vaccine doses also enhanced SARS-CoV-2 S-specific IgA levels above the threshold in plasma from all naïve and pre-infected HCWs but significantly higher IgA concentrations were detected in previously-infected compared to naïve individuals. Furthermore, a significant moderate correlation was observed between S-specific IgA in oral fluid and plasma post-2^nd^ dose from pre-infected individuals (S: spearman r=0.5753, p=0.0064) (**Supplementary Figure 8**).

### COVID-19 mRNA vaccination drives mucosal neutralizing responses to variants of concerns in oral fluid

**Figure 6:**
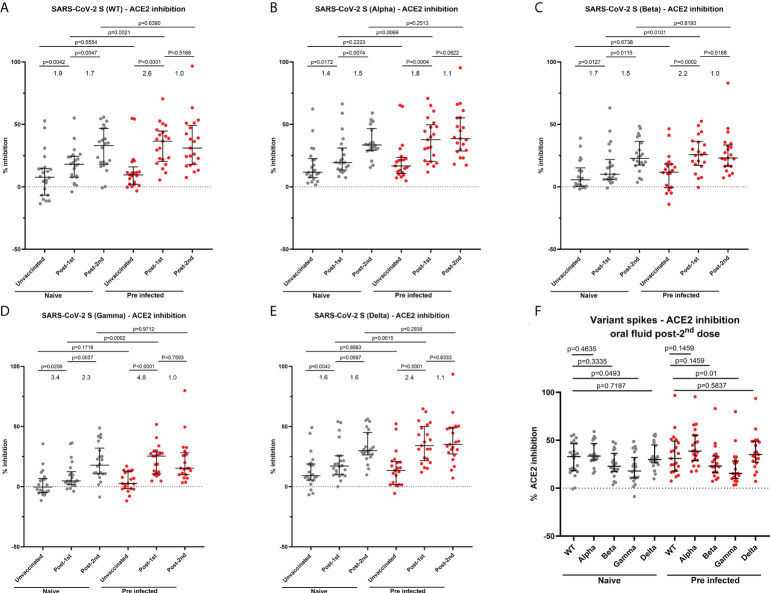
Ability of oral fluid samples to inhibit ACE2 binding to different variants of SARS-CoV-2 spike. **(A–E)** Inhibition of ACE2 binding to SARS-CoV-2 spike Wuhan (WT), B 1.1.7 (Alpha) **(B)**, B 1.351 (Beta) **(C)**, P.1 (Gamma) **(D)** and B 1.617.2 (Delta) **(E)** by matched oral fluid from naïve and previously-infected individuals determined using MSD^®^ ACE2 inhibition assay. **(F)** Comparison of ACE2 binding inhibition to SARS-CoV-2 variant spike antigens post-2^nd^ dose. Wilcoxon and Mann-Whitney tests were used to determine the statistical significance between the groups of paired and unpaired samples, respectively.

Given the critical role of neutralizing antibodies in protection, we evaluated neutralizing responses to Wuhan strain (WT) and an array of VOC (Delta, Alpha, Beta and Gamma) in oral fluids using the MSD^®^ ACE2 inhibition assay, as a surrogate for virus neutralization. Before vaccination, there was no difference in oral fluid neutralizing responses between naïve and previously-infected HCWs (**Figures 6A–E**). In naïve participants, we observed a significant 1.9-fold increase in mucosal neutralizing responses to the Wuhan strain and 1.4 to 3.4-fold increases in neutralizing responses to all VOC following the first vaccine dose (**Figures 6A–E**). A second dose further enhanced mucosal neutralizing responses to the Wuhan strain (1.7-fold change) and all VOC (1.5-2.3-fold change) (**Figures 6A–E**). In previously-infected individuals, a first vaccine dose led to a 2.6-fold increase in mucosal neutralizing responses to the Wuhan strain and 1.8-4.8-fold increases in neutralizing responses to VOC (**Figures 6A–E**). A second vaccine dose did not further enhance mucosal neutralizing responses of previously-infected individuals. Following the first dose, previously-infected individuals generated significantly higher neutralizing responses to Wuhan strain and all VOC compared to naïve individuals. However, no significant differences in neutralizing response magnitude were detected between naïve and previously-infected individuals following the second dose (**Figures 6A–E**). Following 2 vaccine doses, mucosal neutralizing responses were lowest against Beta and Gamma variants (**Figure 6F**).

A weak but significant correlation was observed between Wuhan S-specific ACE2 inhibition and S-specific IgG levels in naïve HCWs (r=0.4234, p=0.0003) (**Supplementary Figure 9A**). In previously-infected individuals, moderate and weakly significant correlations were reported between Wuhan S-specific ACE2 inhibition and IgG (r=0.5642, p<0.0001) (**Supplementary Figure 9B**), as well as IgA responses (r=0.4545, p=0.0001) (**Supplementary Figure 9C**), respectively. The correlation between mucosal IgG concentrations and ACE2 inhibition was greater in previously-infected individuals (**Supplementary Figure 9B**). Similar correlations were observed between RBD-specific IgG levels and the percentage of ACE2 inhibition in naïve (**Supplementary Figure 9D**) and previously-infected participants (**Supplementary Figure 9E**), respectively. However, the correlation between RBD-specific IgA levels and ACE2 inhibition was weaker compared with the correlation between S-specific IgA levels and ACE2 inhibition in previously-infected HCWs (**Supplementary Figure 9F**).

### A breakthrough infection or a third mRNA vaccine dose enhances mucosal antibody responses

**Figure 7:**
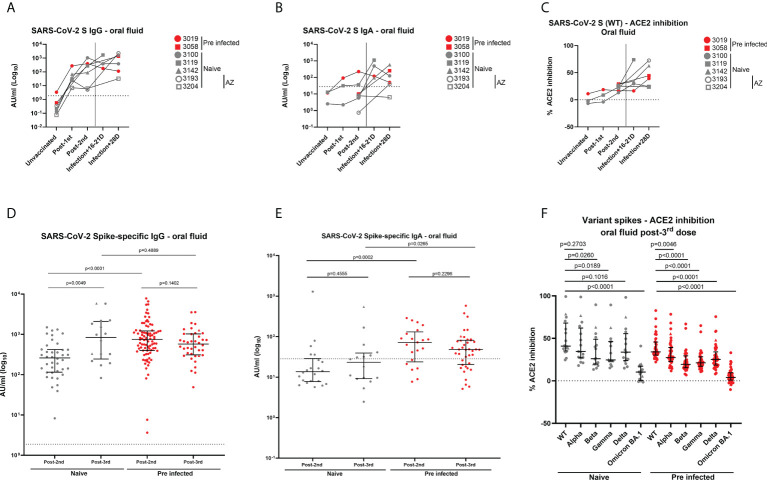
Antibody levels in oral fluid and ability of oral fluid to inhibit ACE2 binding to SARS-CoV-2 spike after a breakthrough infection or a 3^rd^ dose of mRNA vaccine. **(A)** IgG concentrations (AU/ml) and **(B)** IgA concentrations (AU/ml) determined in oral fluid samples from five naïve and 2 previously-infected individuals 16-28 days after breakthrough infections using MSD^®^ immunoassay. **(C)** Inhibition of ACE2 binding to Wuhan SARS-CoV-2 spike (WT) by oral fluid samples from above-mentioned naïve and previously-infected individuals 16-28 days after breakthrough infections determined using MSD^®^ ACE2 inhibition assay. **(D)** IgG concentrations (AU/ml) and **(E)** IgA concentrations (AU/ml) determined in oral fluid from naïve and previously-infected HCWs 28 days post-2^nd^ dose and 28 days post-3^rd^ dose using MSD^®^ immunoassay. **(F)** Comparison of ACE2 binding inhibition to SARS-CoV-2 variant spike antigens (WT, Alpha, Beta, Gamma, Delta, Omicron BA.1) post-3^rd^ dose. Wilcoxon and Mann-Whitney tests were used to determine the statistical significance between the groups of paired and unpaired samples, respectively. **(D–F)** Triangles show the individuals who experienced a breakthrough infection between the 2^nd^ and 3^rd^ dose.

Five individuals among our cohort vaccinated with two doses of BNT162b2 experienced a breakthrough infection between July and September 2021. In order to increase the number of double-vaccinated individuals who were infected at that time, we included in this specific analysis two participants who were naïve at baseline and vaccinated with two doses of AstraZeneca at the time of breakthrough infection (3193: 26 years old, female, 63-day dose interval; 3204: 63 years old, female, 80-day dose interval). S-specific IgG responses in oral fluid were enhanced in four of five naïve individuals and one of two previously-infected individuals following a breakthrough infection (**Figure 7A**). A similar trend was observed for S-specific IgA responses early post-infection. (**Figure 7B**). Interestingly, we also detected an enhancement of neutralizing responses in oral fluid from all individuals following breakthrough infections except in oral fluid from one naïve individual (**Figure 7C**).

We also analyzed the impact of a 3^rd^ mRNA vaccine dose on antibody responses in oral fluid samples from 16 naïve and 40 pre-infected HCWs from our initial cohort vaccinated with two doses of BNT162b2. All participants received BNT162b2 as 3^rd^ dose except two HCWs who received mRNA-1273. We measured IgG, IgA and neutralizing responses against VOC, including Omicron BA.1 28 days post-3^rd^ dose (IQR 27-43). A 3^rd^ dose of mRNA vaccine led to similar range of IgG levels measured post-2^nd^ dose in naïve and previously-infected individuals, respectively. However, significantly higher IgG levels were detected in naïve participants post-3^rd^ dose compared to post-2^nd^ dose (**Figure 7D**, **Supplementary Figure 10A**). An opposite trend was observed in previously-infected individuals (**Figure 7D**). This trend was shown to be significant in matched oral fluid samples from previously-infected HCWs (**Supplementary Figure 10A**). The difference in IgG levels between naïve and previously-infected individuals observed following the 2^nd^ dose was lost after the 3^rd^ dose (**Figure 7D**; **Supplementary Figure 10A**). Similar to our observations post-2^nd^ dose, IgA responses were especially detected in previously-infected individuals (71%) compared to naïve individuals (44%) (**Figure 7E**). No significant differences were observed in IgA responses after the 2^nd^ dose and the 3^rd^ dose in previously-infected individuals (**Figure 7E**, **Supplementary Figure 10B**). A 3^rd^ vaccine dose also induced robust neutralizing responses against SARS-CoV-2 WT spike, as well as against VOC spike proteins to a lesser extent, as observed post-2^nd^ dose (**Figure 7F**). Neutralizing responses were significantly higher post-3^rd^ dose compared to post-2^nd^ dose in matched oral fluid samples from naïve individuals (**Supplementary Figure 10C**). Following the 3^rd^ vaccine dose, mucosal neutralizing responses were lowest against Beta, Gamma and especially Omicron BA.1 spike antigens (**Figure 7F**).

## Discussion

SARS-CoV-2 licensed vaccines, including BNT162b2, are efficacious for preventing severe disease leading to hospitalization and death (20), even though boosters may be required to maintain a high effectiveness against infection with VOC (21–23). Several studies also reported decreased transmission of SARS-CoV-2 among vaccinated people especially early post-vaccination (24). However, the effectiveness against transmission of VOC is more variable (25) due to the circulation of more antigenically distant and transmissible variants (26). In addition, the current approved SARS-CoV-2 vaccines are administered intramuscularly which may not reflect the most efficient route to induce protective immunity at mucosal surfaces (27). Characterizing the nature of protective mucosal immunity against SARS-CoV-2 is essential to improve the effectiveness of the second-generation vaccines against transmission.

In this study, we performed a comparative analysis of SARS-CoV-2 humoral responses before and after BNT162b2 mRNA vaccination in oral fluid and plasma from 200 HCWs who were either naïve or SARS-CoV-2 previously-infected. We analyzed the impact of previous SARS-CoV-2 infection on the nature and kinetics of mucosal and systemic antibody responses. First, we found that the discrimination between seronegative and seropositive individuals before vaccination was feasible using concentrations of IgG in oral fluid. However, the sensitivity was slightly lower in oral fluid compared to plasma using MSD^®^ multiplex assay. The discrepancies between IgG levels in oral fluid and plasma observed in some individuals could suggest a more rapid decay of mucosal IgG responses compared to systemic responses, a difference in mucosal and systemic response induction or in transudation of IgG into mucosal surfaces in some individuals. Secondly, we have found that intramuscular mRNA vaccination induced SARS-CoV-2 S- and RBD-specific IgG responses in oral fluid. The kinetics of mucosal and systemic vaccine-induced IgG responses were similar in naïve and previously-infected participants, respectively. Previously-infected individuals generated higher S- and RBD-specific IgG responses to SARS-CoV-2 in oral fluid following vaccination compared to naïve individuals. Strong correlations between SARS-CoV-2 IgG responses in oral fluid and plasma confirmed that infection- and vaccine-induced SARS-CoV-2-specific IgG can access mucosal surfaces from the periphery, probably through transudation. Large-scale, repeated and accurate testing of the population is needed to measure the persistence of SARS-CoV-2 antibodies. It is crucial to monitor antibody responses to detect waning of vaccine- or infection-induced antibody responses and for understanding the immune factors which may be linked to vaccine breakthrough. The simple, rapid non-invasive method of oral fluids/saliva sampling offers obvious advantages to serum/plasma collection. We confirm that oral fluids may be used to evaluate SARS-CoV-2 IgG levels using MSD^®^ multiplex assay. The MSD^®^ multiplex assay is more sensitive than a commercial anti-spike total antibody assay which detected salivary antibodies in previously-infected individuals only 30 days after the second BNT162b2 dose but not at pre-vaccination stage or following one vaccine dose (28).

The MSD^®^ assay detected cross-reactive IgG responses to SARS-CoV-1 and MERS but not cross-reactive IgA responses in oral fluids (data not shown). Cross-reactivity might be associated more with IgG than IgA or could be due to the more rapid decay of mucosal IgA responses compared to IgG (12). Enhancement of cross-reactive mucosal IgG responses to beta coronaviruses were also observed in oral fluid in previously-infected individuals following one vaccine dose.

In our study, we showed that SARS-CoV-2 mRNA vaccination induced a detectable level of S- and RBD-specific IgA responses in a significant proportion of previously-infected individuals. Similar results were not observed in HCWs without pre-existing SARS-CoV-2 immunity. Sano et al. also reported mRNA vaccination induced a weak mucosal SIgA response in naïve individuals, while SIgA induction after vaccination was more efficient in previously-infected individuals (29). Our results suggest that naturally acquired immunity results in mucosal IgA responses which may be reactivated and systemic IgA responses which may be enhanced by vaccination. Using the MSD^®^ multiplex assay, we were not able to discriminate between monomeric IgA and dimeric/polymeric SIgA. However, given the significant correlation between mucosal IgG and IgA responses, as well as between IgA levels in oral fluid and plasma in previously-infected individuals, it may be hypothesized that IgA might also have crossed from the peripheral circulation following each vaccine dose.

Mucosal IgG and IgA binding studies demonstrated differential antibody responses to mRNA vaccination in naive and previously-infected HCWs. However, it was essential to understand the neutralizing ability of mucosal antibody responses in naïve and previously-infected HCWs. Prior to vaccination, we did not detect any difference in neutralizing antibody responses between naïve and previously-infected participants. Individuals included in our study experienced mild or asymptomatic COVID-19. In addition, the median interval between SARS-CoV-2 infection and baseline sampling was 216 days. Waning of mucosal neutralizing responses may explain we were not able to measure neutralizing responses at baseline. Following 2 doses of mRNA vaccine, we observed neutralizing antibody responses of similar magnitude in oral fluid from naïve and previously-infected HCWs. These results are not fully aligned with the findings published by Azzi et al. who observed that neutralizing antibodies were present to a greater extent in previously-infected participants compared to naïve individuals after 2 vaccine doses (18). However, they used an alternative surrogate neutralization assay and we have confirmed a strong correlation between our MSD^®^ ACE2 inhibition assay and live virus neutralization test in several prior studies (4, 5, 30). Other studies have shown that there was a difference in neutralizing responses post-infection where salivary IgA dominated the early neutralizing antibody responses (15) and post-vaccination where salivary IgG strongly correlated with neutralizing responses at least in naïve participants (18). The neutralization activity may be dominated by IgG responses in naïve individuals. However, the positive correlations observed between SARS-CoV-2 S-specific IgG/IgA levels and ACE2 inhibition in our study suggest that both IgG and IgA responses can be partially neutralizing in previously-infected individuals.

We also observed that a breakthrough infection can induce SARS-CoV-2 S-specific IgA in some individuals vaccinated with BNT162b2 vaccination. Interestingly, Sheikh-Mohamed et al. reported that people who experienced a breakthrough infection had lower levels of S- and RBD-specific IgA in serum 2-4 weeks following a second mRNA vaccine dose compared to exposed but not infected control individuals (31). In our cohort, five individuals experienced a breakthrough infection after 2 BNT162b2 doses. In addition, ten individuals from our cohort vaccinated with BNT162b2 were also exposed to an infected household contact but were not infected themselves. However, we did not find a correlation with mucosal IgA levels and breakthrough infection (data not shown). We also showed that a 3^rd^ vaccine dose differentially enhanced mucosal responses in naïve and previously-infected individuals. The benefit of the 3^rd^ dose to further enhance IgG responses was shown to be more significant in naïve compared to previously-infected individuals. In addition, IgA responses were more frequently detected following the 3^rd^ dose in previously-infected individuals compared to naïve HCWs, suggesting a reactivation of mucosal IgA responses by the 3^rd^ dose. As previously described in sera (32, 33), the neutralizing ability of oral fluids from naïve and previously-infected HCWs against VOC was reduced compared to WT spike antigen especially against Omicron BA.1 variant.

Some limitations of our study can be highlighted. Firstly, our cohort was composed of HCWs which led to a predominance of females of working age. Furthermore, our sample size was determined by feasibility of recruitment rather than a pre-determined estimation of statistical power, and therefore our results should be interpreted with caution. Although we had established thresholds for positivity for SARS-CoV-2 antigens using pre-pandemic samples, the same was not possible for the seasonal coronavirus spike proteins due to extensive exposures to these viruses in most adults. Our study also focused on antibody responses given serology is an easy way to analyze immune responses in mucosal fluid. The analysis of innate and cellular responses at respiratory surfaces would further inform knowledge of mucosal immune responses but would require animal models or human tissues/samples we do not have access to. Finally, ongoing follow-up would be needed to evaluate the durability of mucosal neutralizing responses and to include more breakthrough infections.

This study found that vaccine-induced IgG responses strongly correlated in plasma and oral fluid from naïve and previously-infected individuals, respectively. Mucosal neutralizing responses were observed in naïve and previously-infected participants following vaccination. However, mucosal IgA responses were more often detected in previously-infected compared to naïve HCWs. The potential complementary function of mucosal IgG and IgA responses in previously-infected participants may be advantageous but the clinical significance remains to be determined. It is likely that time between a pre-infection and vaccination may also play a role in protection given SARS-CoV-2 S-specific IgA responses in saliva decrease quicker than salivary IgG (12). If a local antigenic stimulation improves the effectiveness of mucosal responses, recurrent exposures post-vaccination associated with a strategy of mucosal boost vaccination, may enhance long-term protective immune responses to SARS-CoV-2.

## Data availability statement

The raw data supporting the conclusions of this article will be made available by the authors, without undue reservation.

## Ethics statement

The studies involving human participants were reviewed and approved by IRAS ID 284460, REC reference 20/SC/0230. The patients/participants provided their written informed consent to participate in this study.

## Author contributions

MC and TS conceptualized the project. SL, AH and SH designed, supervised, performed and analyzed antibody experiments. SH performed the statistical analyzes for **Table 1**. MC and TS provided intellectual expertise. TT provided technical expertise for ACE2 inhibition assays and edited the manuscript. SL wrote the original draft. RB, HH and NM established the clinical cohorts and collected the clinical samples and data. EB, SD, CD, PK, AR and LT edited the manuscript. TS and MC reviewed and edited manuscript and figures. All authors contributed to the article and approved the submitted version.

## Funding

This study was funded by PITCH, NIHR COV19-RECPLAS, NIHR Biomedical Research Centre Oxford, Huo Family Foundation, WT109965MA. EB is supported by the Oxford NIHR biomedical Research Centre and is an NIHR senior investigator. The views expressed in this manuscript do not reflect those of the funding bodies. MC is supported by U.S. Food and Drug Administration Medical Countermeasures Initiative contract (75F40120C00085).

## Conflict of interest

The authors declare that the research was conducted in the absence of any commercial or financial relationships that could be construed as a potential conflict of interest. However, for prior studies, not related to the research published here, MSD have provided access to early phase assay plates free of charge to MC.

## Publisher’s note

All claims expressed in this article are solely those of the authors and do not necessarily represent those of their affiliated organizations, or those of the publisher, the editors and the reviewers. Any product that may be evaluated in this article, or claim that may be made by its manufacturer, is not guaranteed or endorsed by the publisher.
