# Supplementary material for: mRNA vaccination drives differential mucosal neutralizing antibody profiles in naïve and SARS-CoV-2 previously-infected individuals
Source: Front Immunol. 2022 Sep 8;13:953949. doi: 10.3389/fimmu.2022.953949 (PMC9499336; doi:10.3389/fimmu.2022.953949)
Supplement: Supplementary file 1 [file DataSheet_1.docx]

Supplementary Material

# Supplementary Figures and Tables

## Supplementary Figures


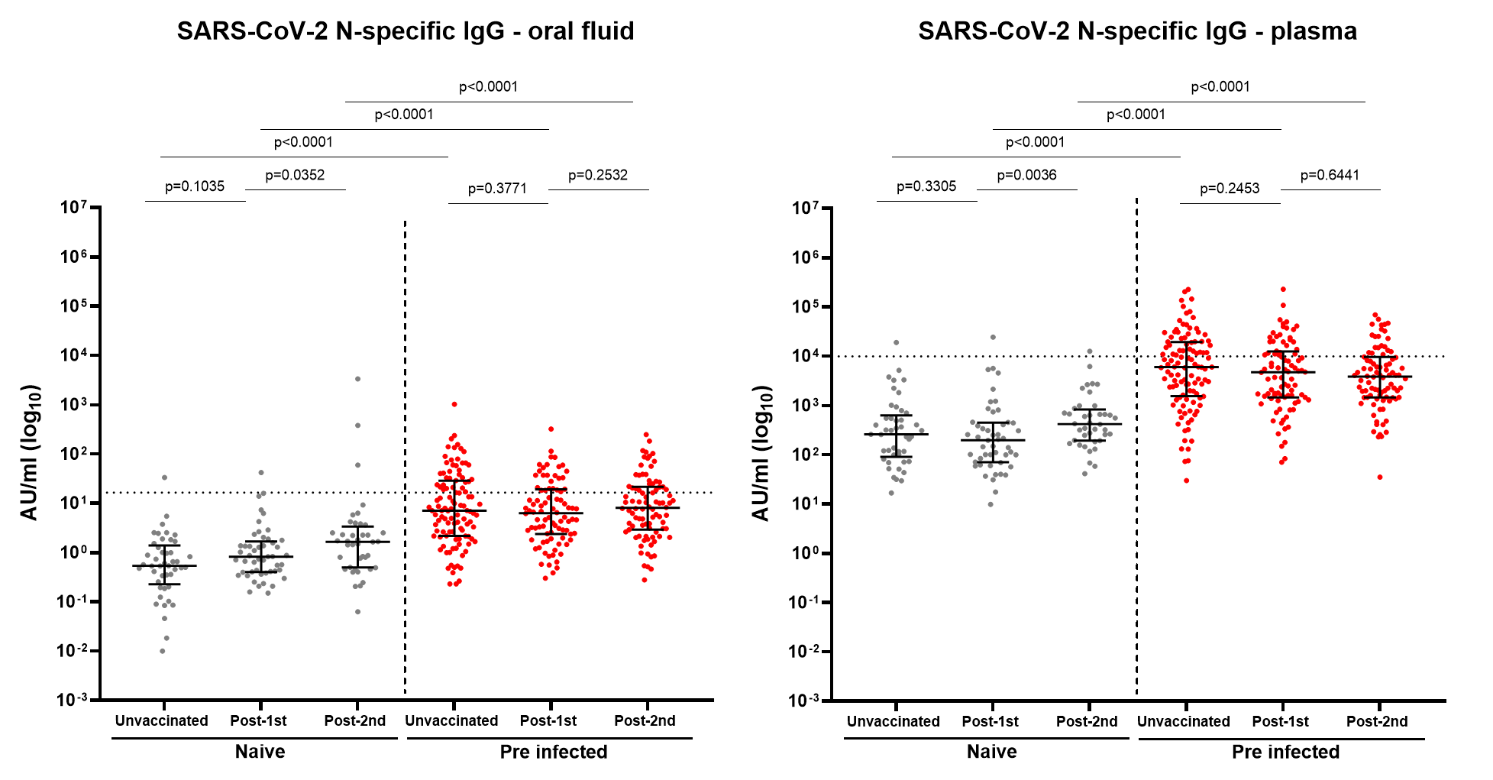


**Supplementary Figure 1:** **SARS-CoV-2 N-specific IgG concentrations in oral fluid and plasma from naïve and previously-infected individuals analyzed by multiplex MSD^®^ assay.** A) N-specific IgG in oral fluid and B) in plasma. Data are shown in concentrations expressed in Arbitrary Units/ml (AU/ml). Mann-Whitney tests were used to determine the statistical significance between the groups of samples. Dashed lines show the cut-offs based on IgG responses in 45 unvaccinated naïve samples (average concentration + 3SD).


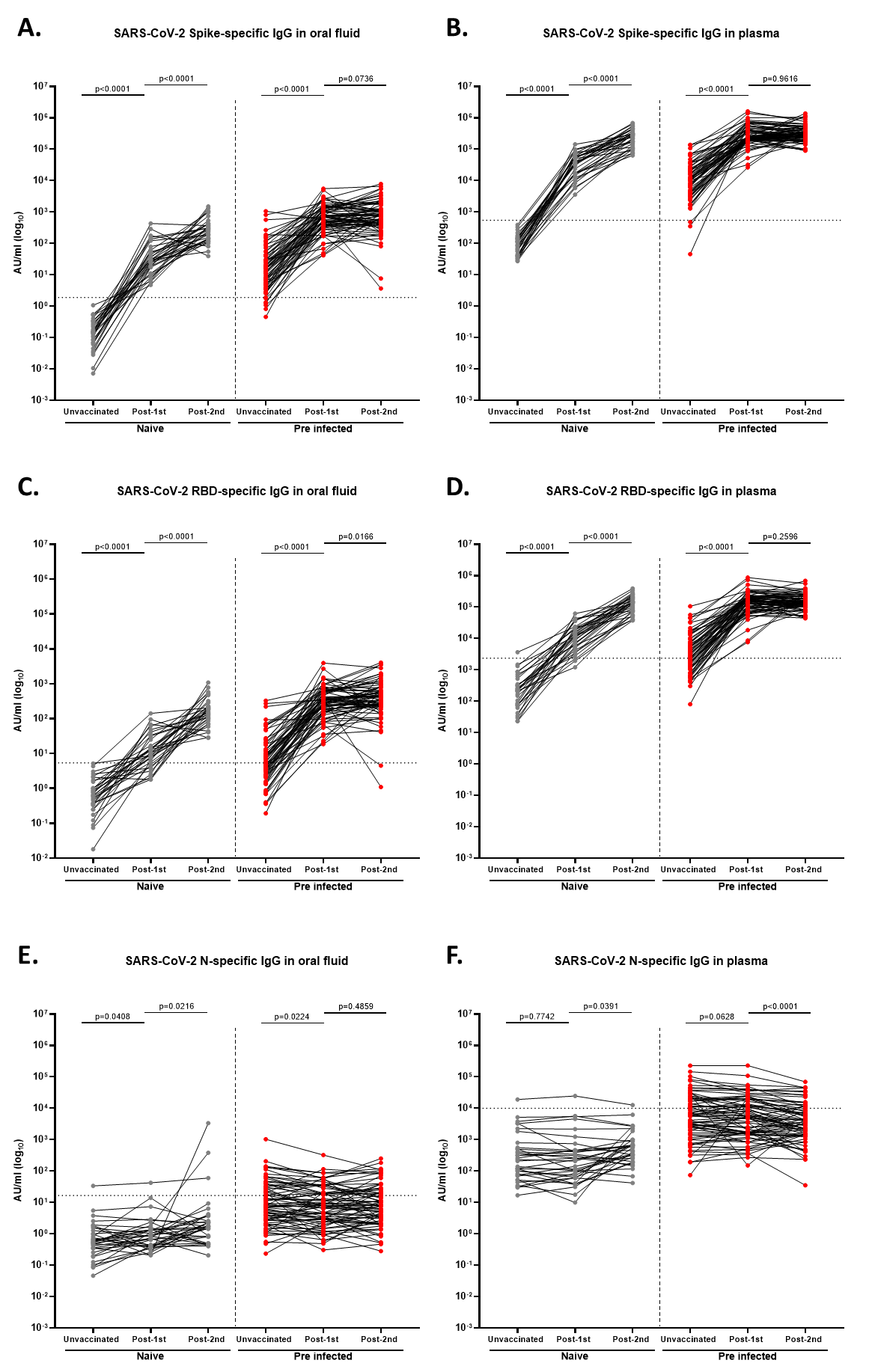


**Supplementary Figure 2:** **SARS-CoV-2-specific IgG concentrations in matched oral fluid and plasma from naïve and previously-infected individuals.** A) S-specific IgG in oral fluid and B) in plasma; C) RBD-specific IgG in oral fluid and D) in plasma; E) N-specific IgG in oral fluid and F) in plasma were measured by MSD^®^ multiplex immunoassay. Data are shown in concentrations expressed in AU/ml. Wilcoxon rank tests were used to determine the statistical significance between the groups of paired samples. Dashed lines show the cut-offs based on IgG responses in 45 unvaccinated naïve samples (average concentration + 3SD). Matched naïve samples: N=34; Matched previously-infected samples: N=74.


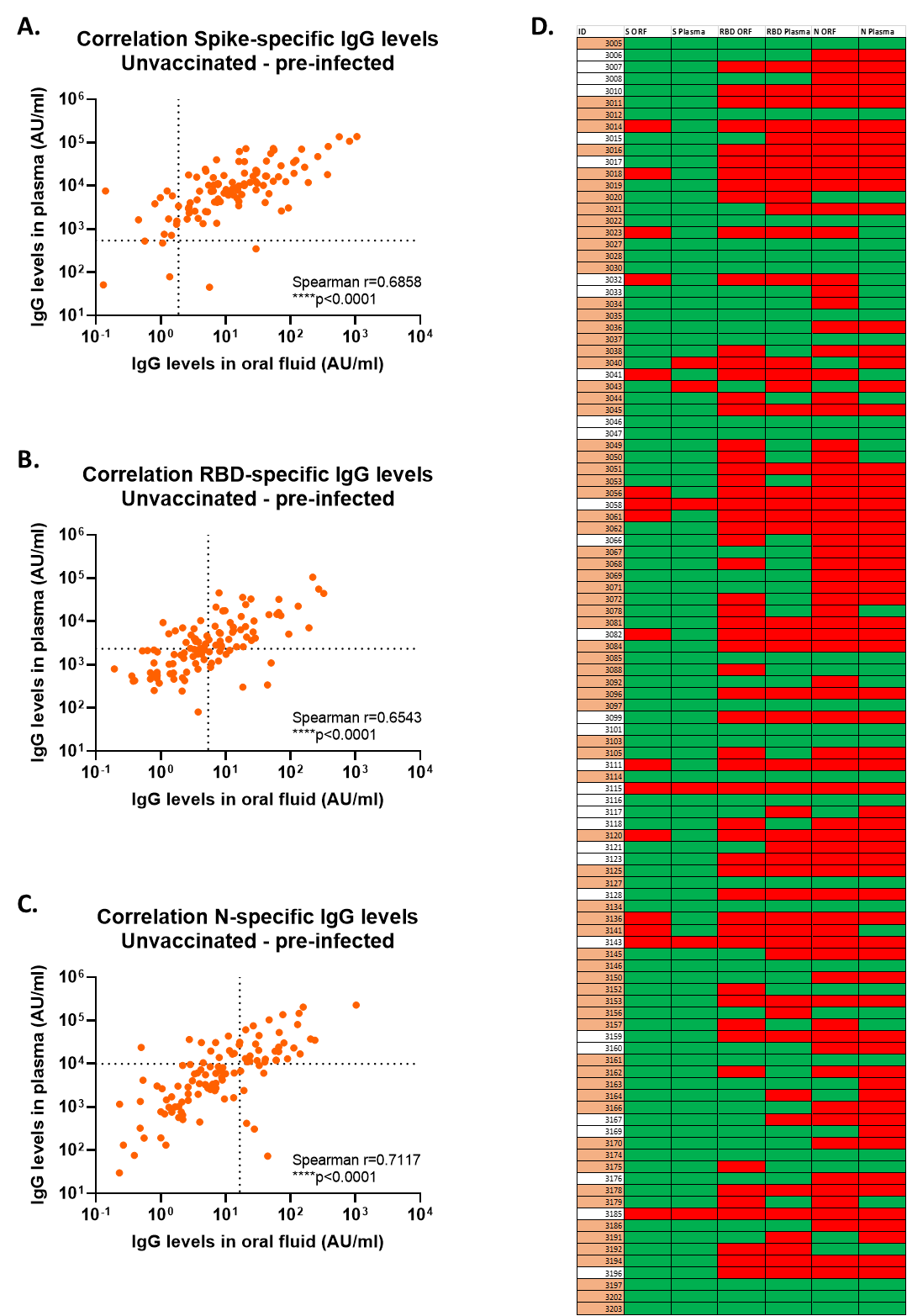


**Supplementary Figure 3:** **Correlations between SARS-CoV-2-specific IgG concentrations in oral fluid and in plasma from SARS-CoV-2 previously-infected individuals before vaccination.** S- (A), RBD- (B) and N-specific (C) IgG concentrations were measured by MSD^®^ multiplex immunoassay. Pairwise correlations were assessed using Spearman's rank-order correlation. D) SARS-CoV-2 seropositivity (green) and seronegativity (red) for S, RBD and N determined in oral fluid (ORF) and plasma from previously-infected HCWs before vaccination. Previously-infected HCWs were defined by PCR and/or NHS serology. The IDs highlighted in orange-brown are those confirmed by PCR test.

**
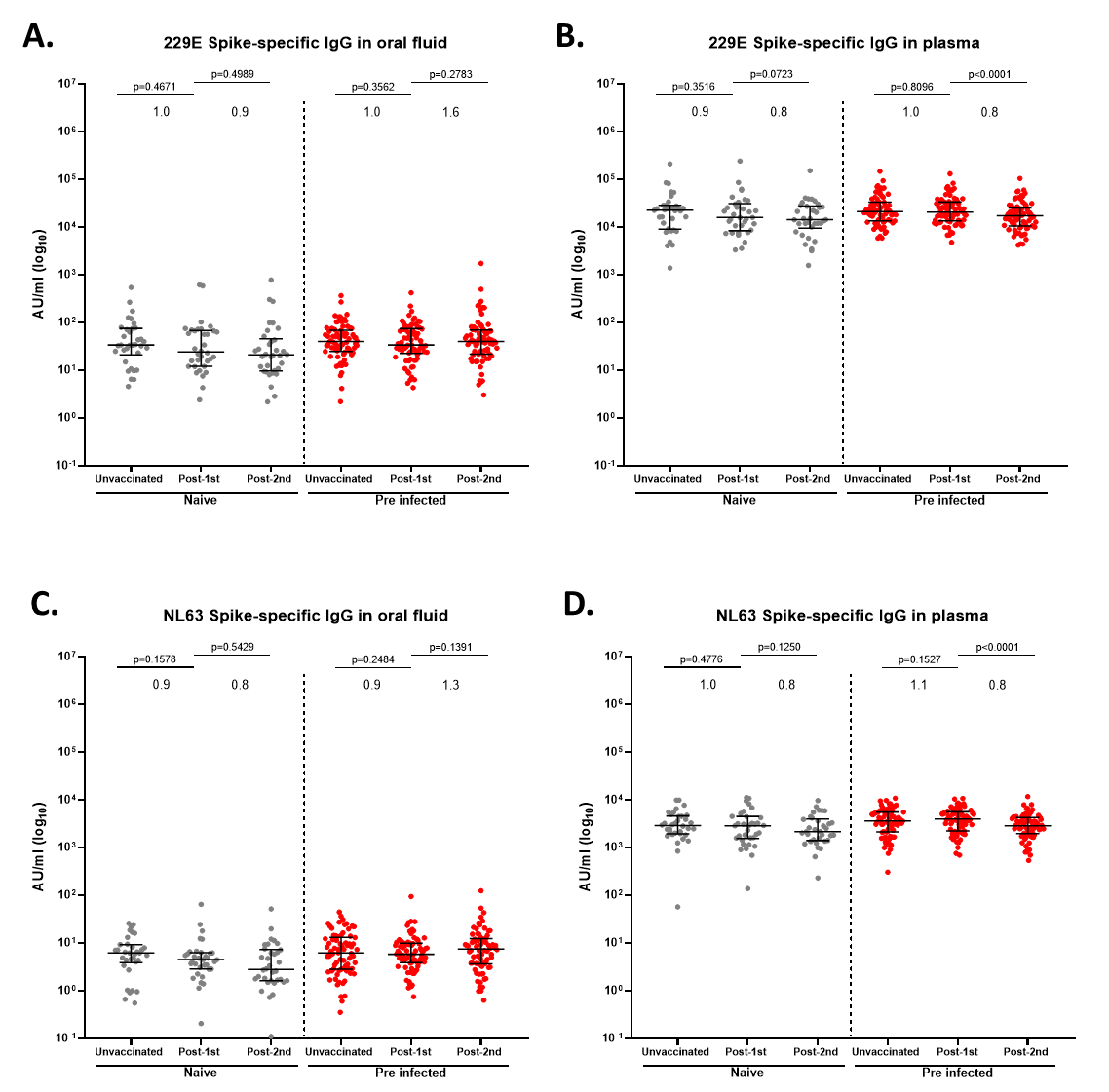
**

**Supplementary Figure 4:** **IgG responses to human seasonal alpha coronaviruses in matched oral fluid and plasma.** Concentrations (AU/ml) of 229E (A & B) and NL63 (C & D) S-specific IgG measured in oral fluid (A & C) and plasma (B & D) from naïve and previously-infected individuals using MSD^®^ multiplex immunoassay. Wilcoxon rank tests were used to determine the statistical differences between paired samples. Values above the columns show the fold increases in S-specific IgG levels. Matched naïve samples: N=34; Matched previously-infected samples: N=74.


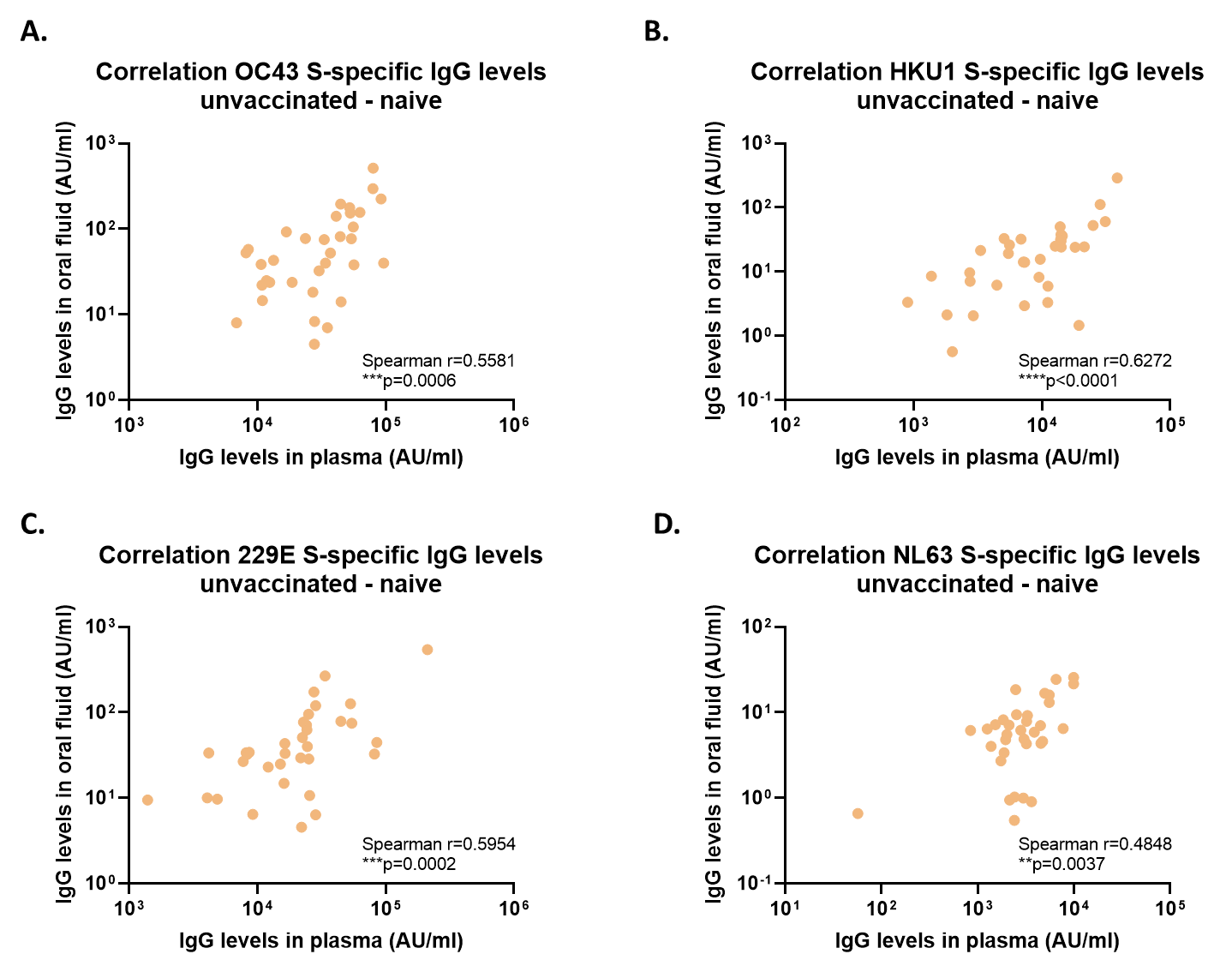


**Supplementary Figure 5: Correlation between seasonal coronavirus IgG levels in oral fluid and plasma from naïve individuals before vaccination.** OC43 (A), HKU1 (B), 229E (C) and NL63 (D) S-specific IgG concentrations (AU/ml) were measured by MSD^®^ multiplex immunoassay. Pairwise correlations were assessed using Spearman's rank-order correlation.


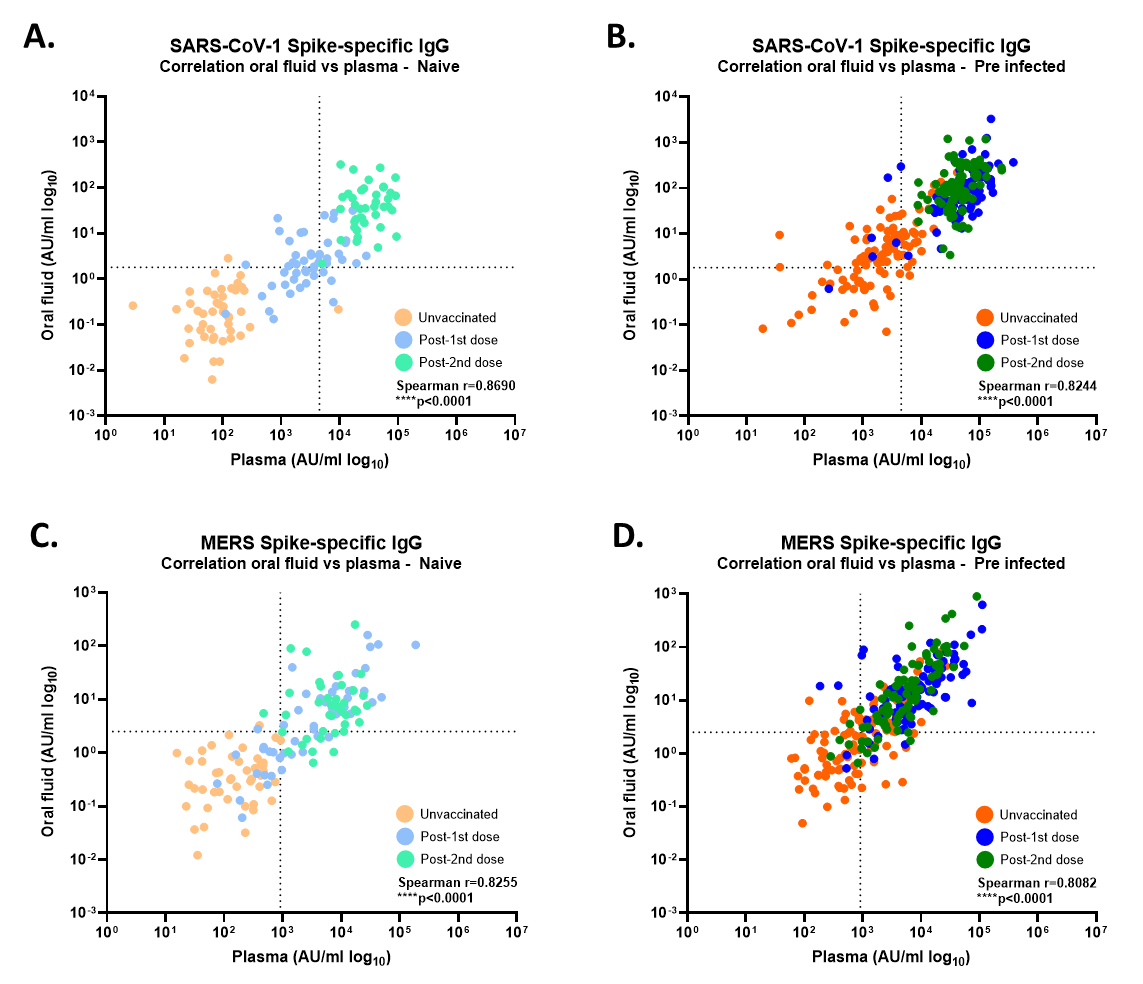


**Supplementary Figure** **6: Correlations between** **IgG responses to SARS-CoV-1 and MERS in oral fluid and plasma.** Correlations between SARS-CoV-1 (A & B) and MERS S-specific (C & D) IgG in oral fluid versus plasma from naïve (A & C) and pre-infected (B & D) individuals. Pairwise correlations were assessed using Spearman's rank-order correlation.

**Supplementary Figure 7: SARS-CoV-2 N-specific IgA response.** SARS-CoV-2 N-specific IgA concentrations (AU/ml) in matched oral fluid samples from naïve and previously-infected individuals determined by multiplex MSD^®^ immunoassay. Wilcoxon tests were used to determine the statistical significance between the groups of paired samples. Dashed lines show the cut-offs based on IgA responses in 22 unvaccinated naïve samples (average concentration +1SD).


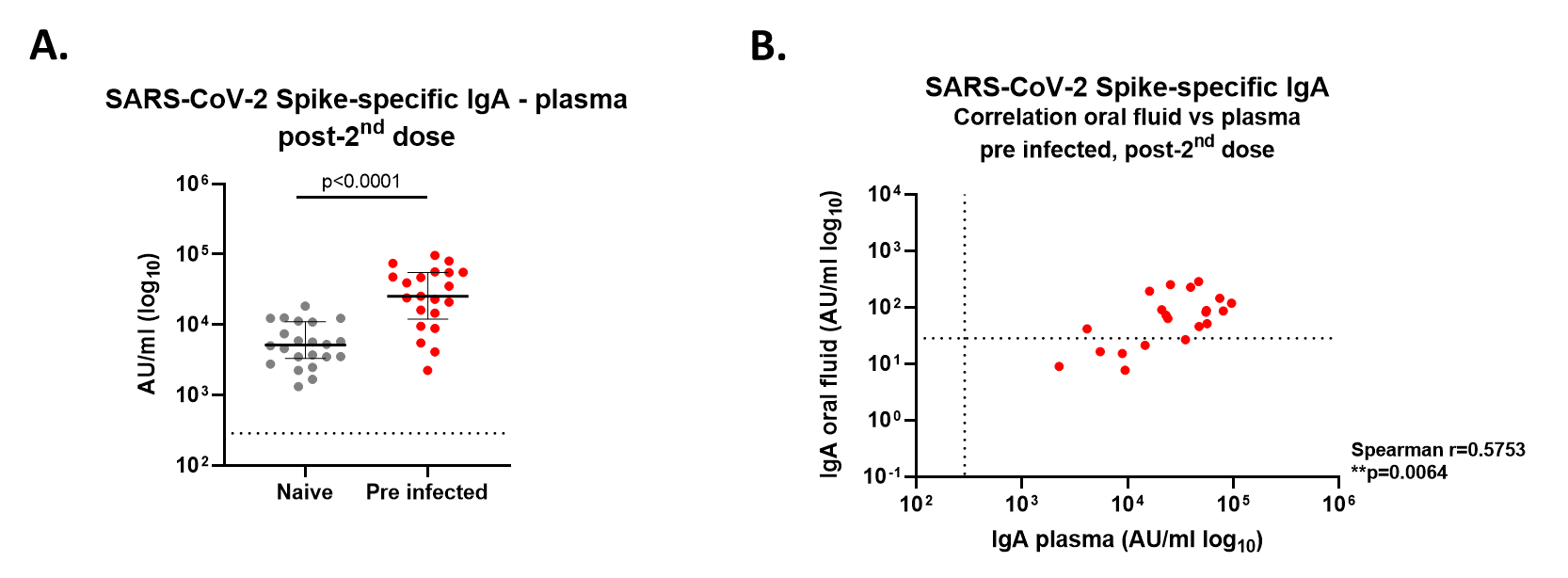


**Supplementary Figure 8: SARS-CoV-2 spike-specific IgA levels in plasma and correlation with IgA levels in oral fluid post-2^nd^ dose.** A) Spike-specific IgA concentrations (AU/ml) in plasma from naïve and previously-infected HCWs post-2^nd^ dose measured by MSD^®^ multiplex immunoassay. Mann-Whitney tests were used to determine the statistical significance between the groups of unpaired samples. B) Correlation between IgA concentrations measured in oral fluid and plasma post-2^nd^ dose in previously-infected individuals. Pairwise correlations were assessed using Spearman's rank-order correlation. Dashed lines show the cut-offs based on IgA responses in 22 unvaccinated naïve samples (average concentration +1SD).

**
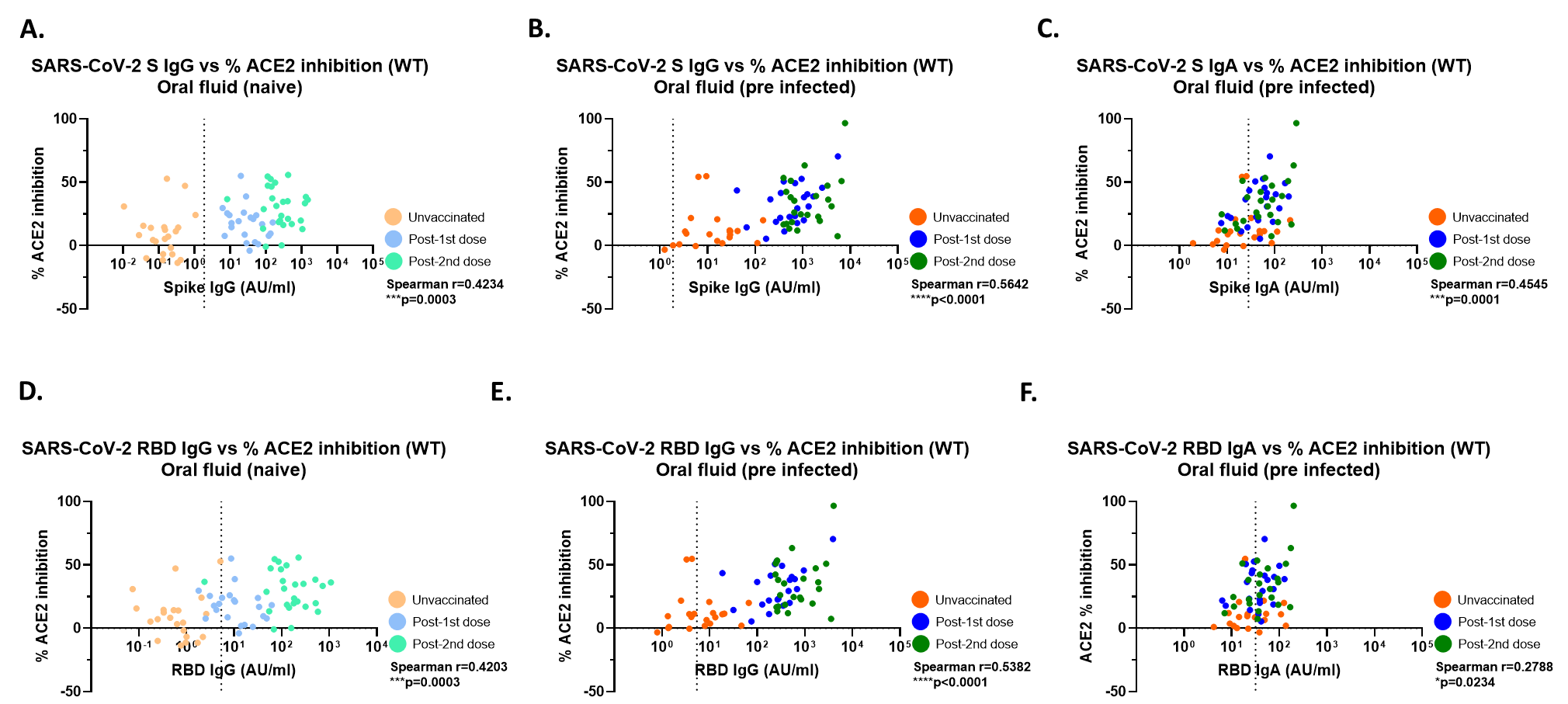
**

**Supplementary Figure 9: Correlations between ACE2 binding inhibition to Wuhan SARS-CoV-2 spike and SARS-CoV-2-specific antibody levels.** Correlations between SARS-CoV-2 S- (A, B & C) or RBD- (D, E & F) specific IgG (A, B, D & E) or IgA (C & F) concentrations and percentage of ACE2 inhibition in oral fluid of naïve (A & D) and previously-infected (B, C, E & F) individuals. Pairwise correlations were assessed using Spearman's rank-order correlation.

**
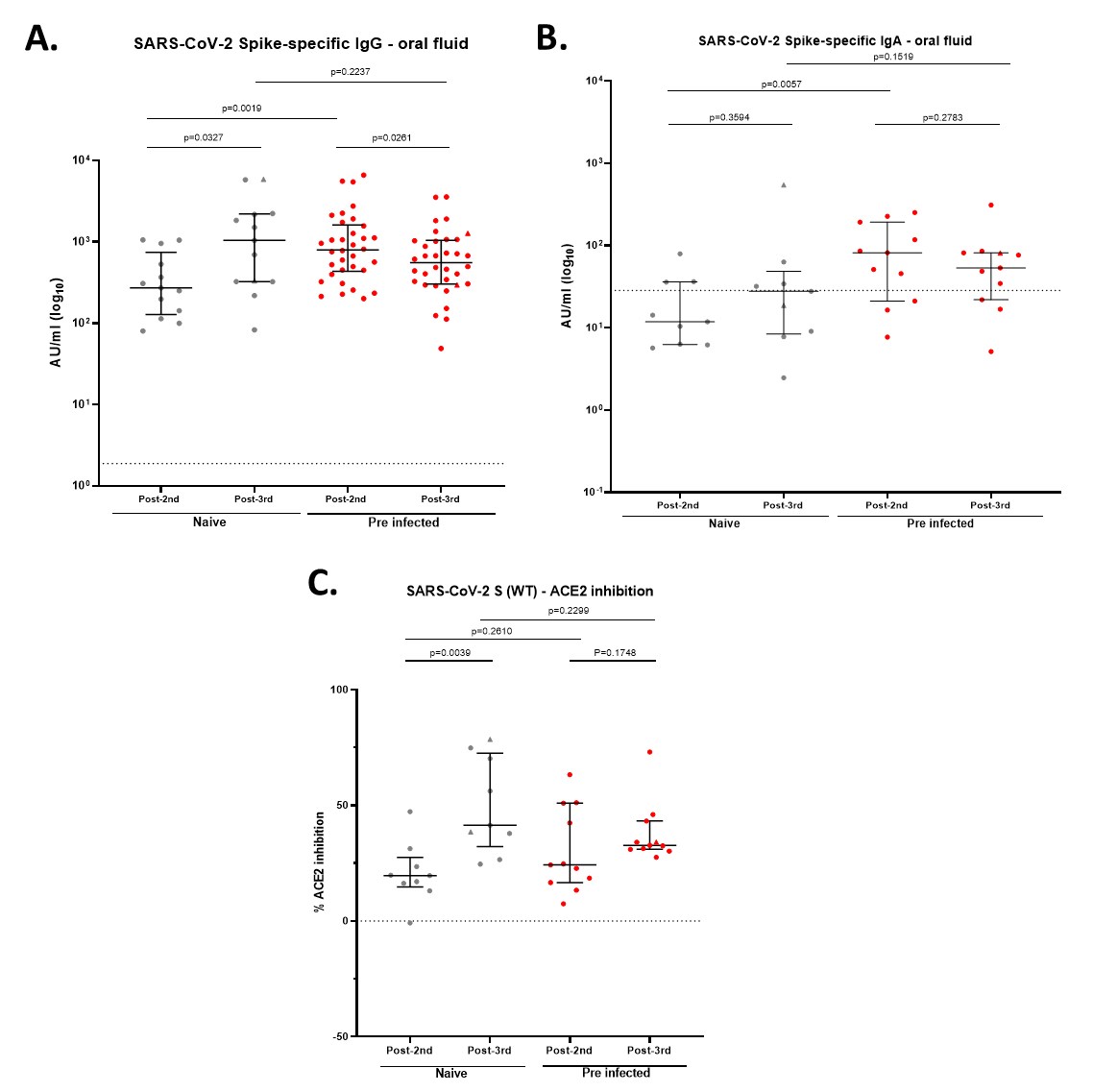
**

**Supplementary Figure 10: Antibody levels in oral fluid and ability of oral fluid to inhibit ACE2 binding to Wuhan SARS-CoV-2 spike after the 2^nd^ dose and the 3^rd^ dose of mRNA vaccine**. A) IgG concentrations (AU/ml) and B) IgA concentrations (AU/ml) determined in matched oral fluid samples 28 days after the 2^nd^ dose and 28 days after the 3^rd^ dose using MSD^®^ immunoassay. C) Inhibition of ACE2 binding to Wuhan SARS-CoV-2 spike (WT) by matched oral fluid samples from naïve and previously-infected individuals determined using MSD^®^ ACE2 inhibition assay. Wilcoxon and Mann-Whitney tests were used to determine the statistical significance between the groups of paired and unpaired samples, respectively. Triangles show the individuals who experienced a breakthrough infection between the 2^nd^ and 3^rd^ dose.

## Supplementary Table

| **Oral fluid** | S-specific IgG | No S-specific IgG | **Sensitivity**  **(95% CI)** | **Specificity**  **(95% CI)** |
| --- | --- | --- | --- | --- |
| Previously-infected | 68 | 8 | 0.89  (0.81-0.95) | 0.98  (0.88-1.00) |
| Naïve | 1 | 44 |  |  |
| **Plasma** | S-specific IgG | No S-specific IgG | **Sensitivity**  **(95% CI)** | **Specificity**  **(95% CI)** |
| Previously-infected | 74 | 2 | 0.97  (0.91-1.00) | 0.98  (0.88-1.00) |
| Naïve | 1 | 44 |  |  |

**Supplementary Table 1:** **Sensitivity and specificity of MSD^®^ immunoassay with 95% confidence intervals using only previously-infected HCWs confirmed by PCR test.** Analysis performed using S-specific IgG responses measured in oral fluid and plasma samples from pre-infected HCWs confirmed by PCR test and naïve individuals at pre-vaccination stage. Previously-infected individuals N=76, Naïve individuals N=45.

**
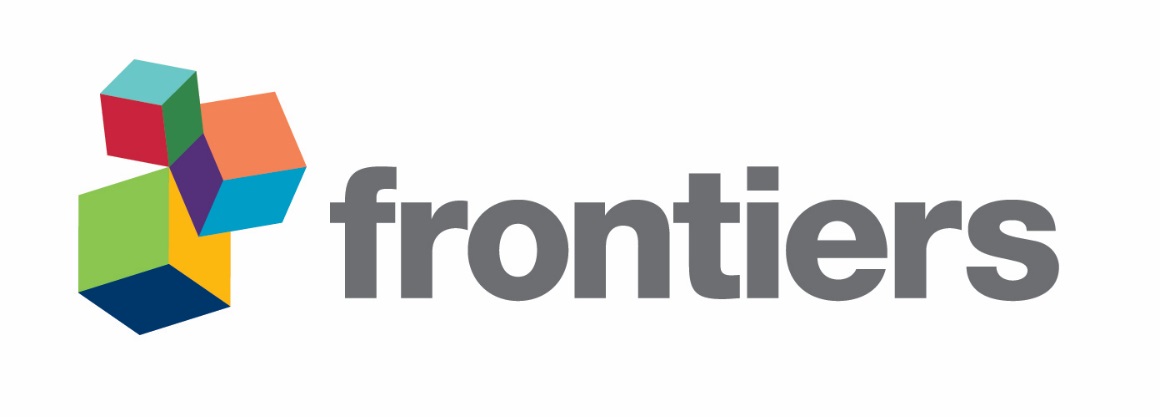
**
